# Intrapulmonary course of the inferior vena cava: a rare congenital anomaly

**DOI:** 10.1093/bjrcr/uaag017

**Published:** 2026-04-24

**Authors:** Pietro Sergio, Daniele Distefano, Riccardo De Marchi, Ilaria Zangrandi, Giorgia Maretto, Giulia D’incerto, Salvatore Golemi, Tiziana Guarneri, Margherita Muri, Pietro Ciolli, Giuseppe Voltini, Lorenza Carnevale, Gianluca Romeo

**Affiliations:** Department of Radiology, ASST Cremona, Cremona 26100, Italy; Department of Radiology, ASST Cremona, Cremona 26100, Italy; Department of Radiology, ASST Cremona, Cremona 26100, Italy; Department of Radiology, ASST Cremona, Cremona 26100, Italy; Department of Radiology, ASST Cremona, Cremona 26100, Italy; Department of Radiology, ASST Cremona, Cremona 26100, Italy; Department of Radiology, ASST Cremona, Cremona 26100, Italy; Department of Radiology, ASST Cremona, Cremona 26100, Italy; Department of Radiology, ASST Cremona, Cremona 26100, Italy; Department of Radiology, ASST Cremona, Cremona 26100, Italy; Department of Radiology, ASST Cremona, Cremona 26100, Italy; Department of Radiology, ASST Cremona, Cremona 26100, Italy; Department of Radiology, ASST Cremona, Cremona 26100, Italy

**Keywords:** Inferior vena cava, Congenital anomalies, CT

## Abstract

Congenital anomalies of the inferior vena cava (IVC) are often encountered incidentally in cross sectional imaging modalities. The most frequently described anomalies include retroaortic left renal vein, left IVC, double IVC, circumaortic left renal vein, interruption of IVC with azygos and hemiazygos continuation, absence of the infrarenal IVC, and circumcaval ureter. This report shows an unusual case of congenital inferior vena cava anomaly, characterized by an intrapulmonary course of the inferior vena cava.

## Introduction

Congenital anomalies of the inferior vena cava (IVC) are often incidentally detected using cross-sectional imaging modalities.

The most frequently observed and published anomalies include the retroaortic left renal vein, left IVC, double IVC, circumaortic left renal vein, interruption of IVC with azygos and hemiazygos continuation, absence of the infrarenal IVC, and circumcaval ureter.^1–6^

We present the case of an abnormal course and anatomical variations of the suprahepatic and suprarenal IVC tract, documented by a chest X-ray and chest CT.

The most significant anomaly identified is the intrapulmonary course of the suprahepatic IVC tract.

To the best of our knowledge, no other cases, featuring the same anomalies, as those described in this report, have ever been published previously.

## Case presentation

A 27-year-old man was referred to our hospital for evaluation after a fall from a bicycle.

He was previously in good health, his vital signs were normal, and his only symptom was pain on the right side.

The X-ray of his right forearm showed fractures of the radius and ulna.

**Figure 1:**
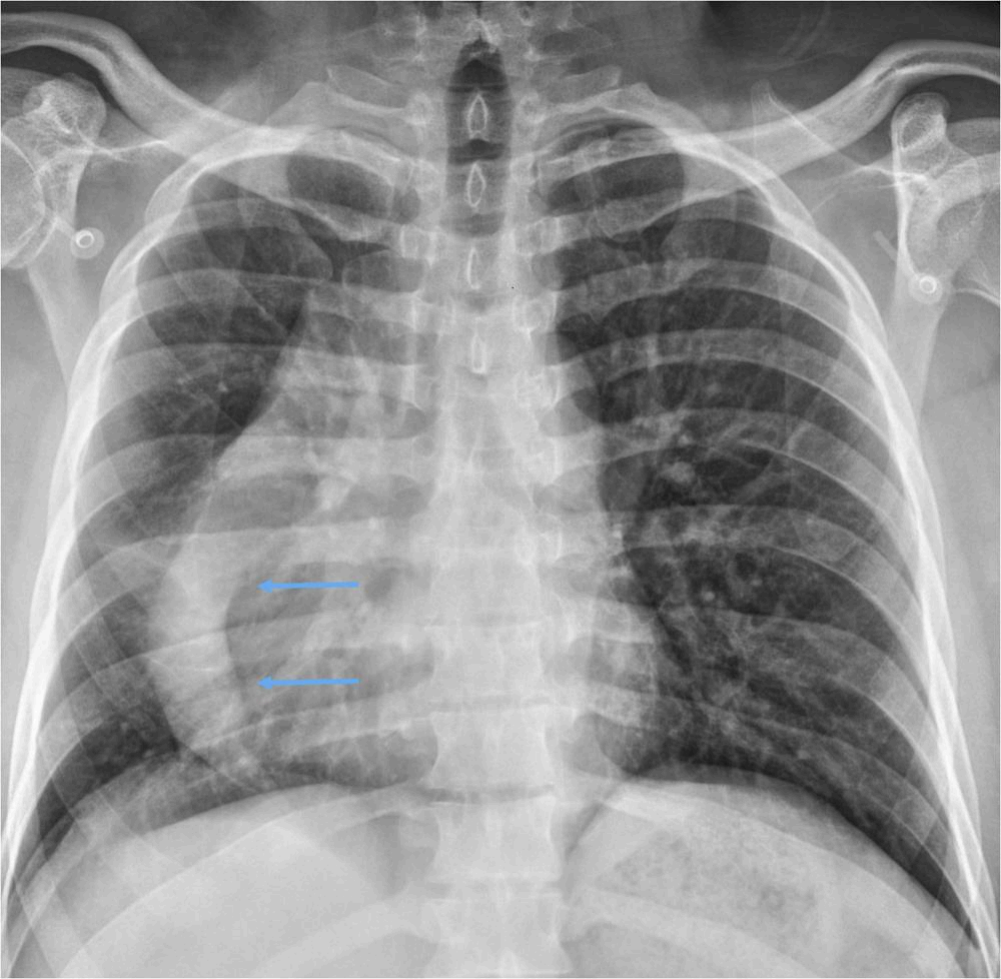
The chest X-ray shows a scimitar-shaped tubular structure in the right lung (arrows) and cardiac dextroposition.

The chest X-ray indicated an anomaly in the right lung, resembling a curved Turkish sword (scimitar), along with dextroposition of the heart (Figure 1).

The patient later underwent a contrast chest CT scan for further evaluation.

The CT scan detected an abnormal course and some anatomical variations of the suprahepatic and suprarenal inferior vena cava.

**Figure 2:**
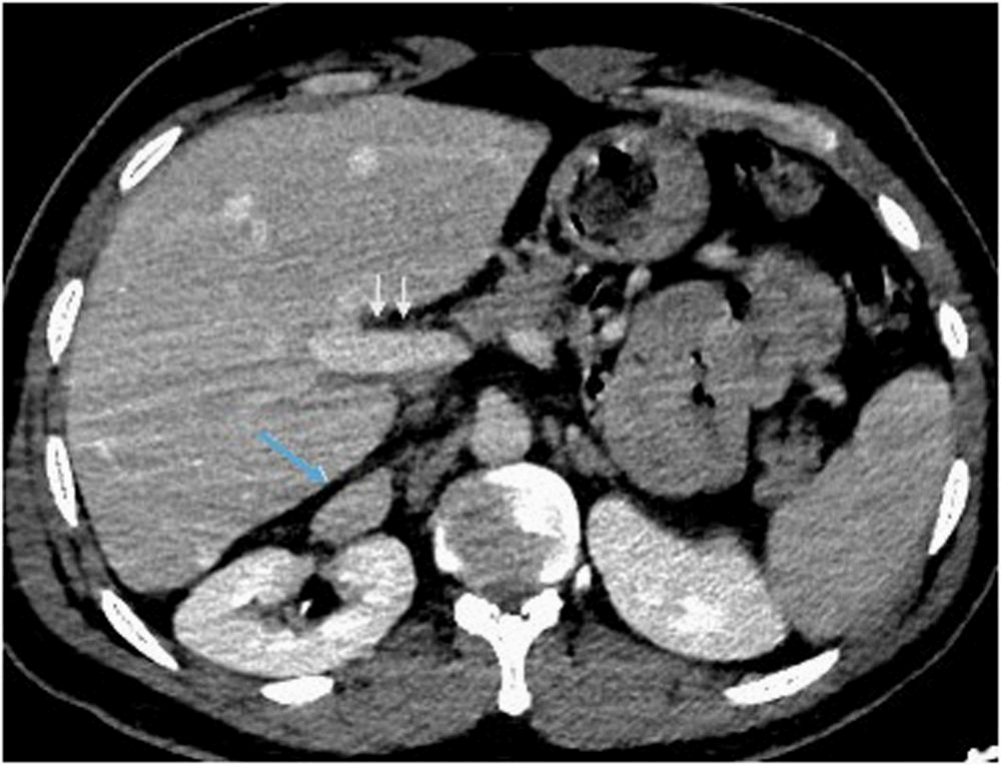
The suprarenal inferior vena cava tract is abnormal in its course, located in the hepatorenal space (arrow). The portal vein is normal (white arrows).

In particular, the suprarenal IVC was not in its usual right periaortic site but appeared to be located in the hepatorenal space, without any passage through the liver (Figure 2).

**Figure 3:**
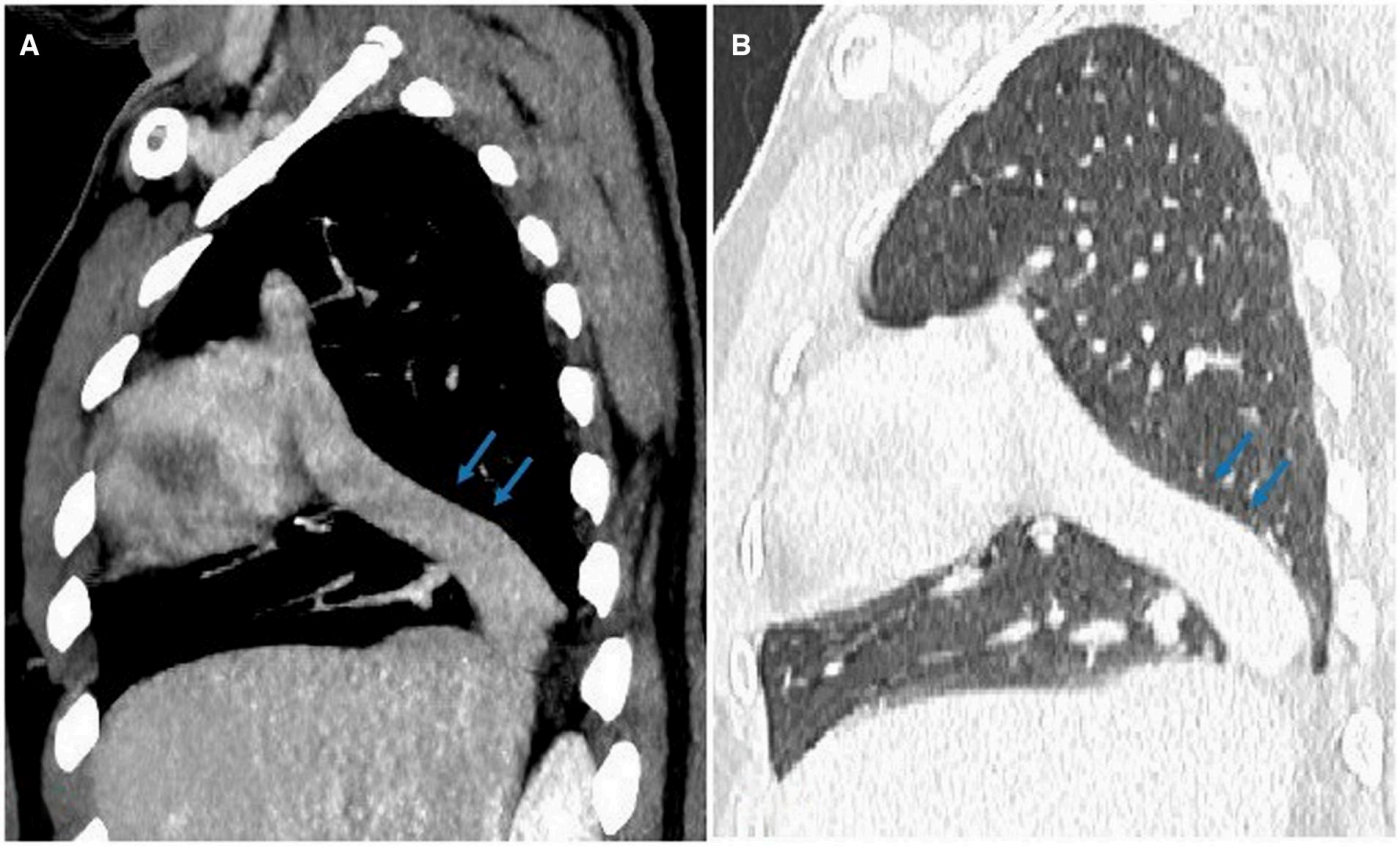
(A) mediastinal window, (B) lung window. The sagittal CT image shows the inferior vena cava crossing the right lung and draining into the right atrium (arrows).

The suprahepatic IVC tract reached the right atrium by passing through the right lung, mimicking the shape of a scimitar (Figure 3A and B).

**Figure 4:**
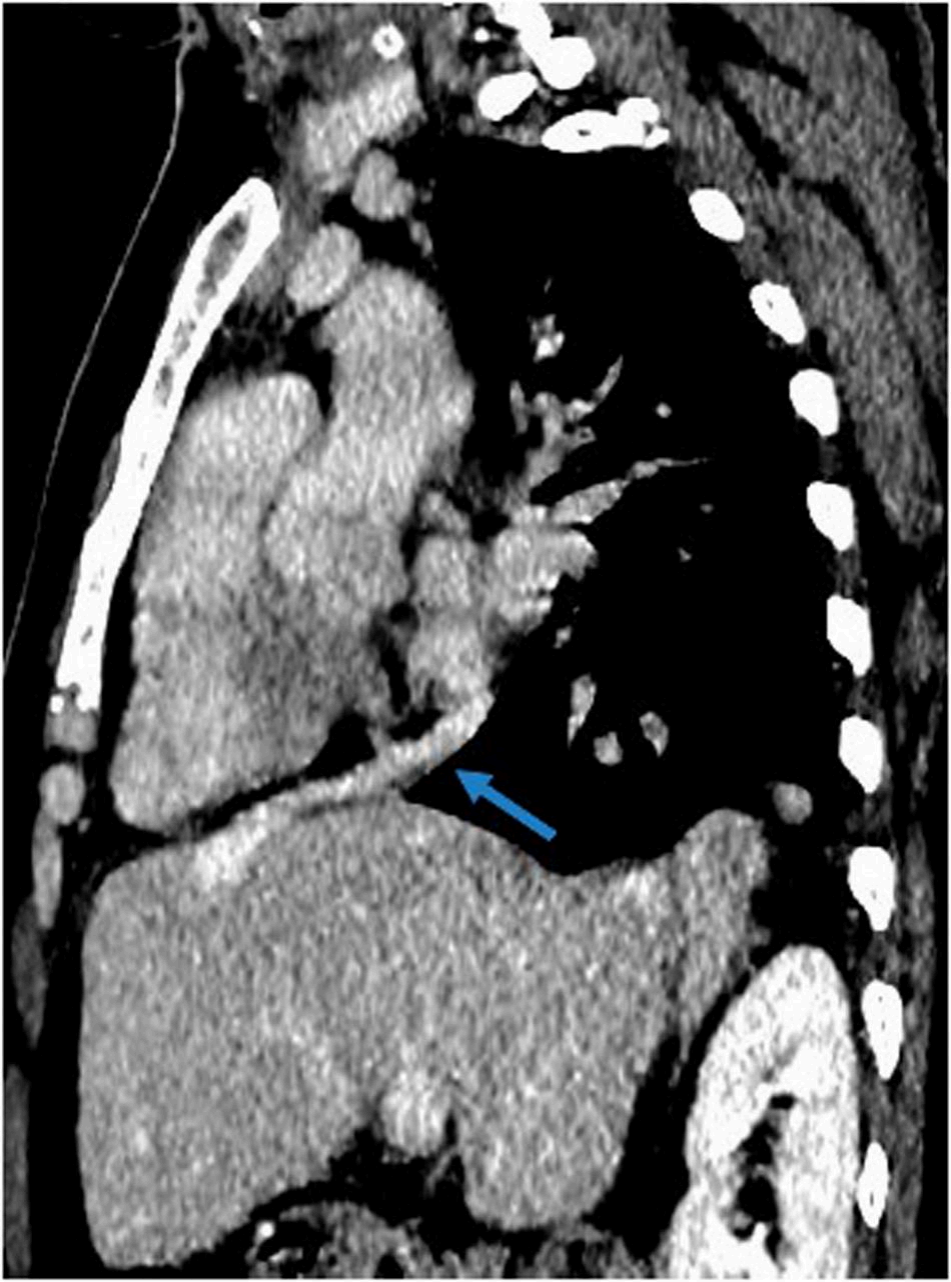
The sagittal CT image shows the venous duct originating from the hepatic veins, reaching the right atrium (arrow).

The hepatic veins gave rise to a common venous duct that drained into the right atrium (Figure 4).

**Figure 5:**
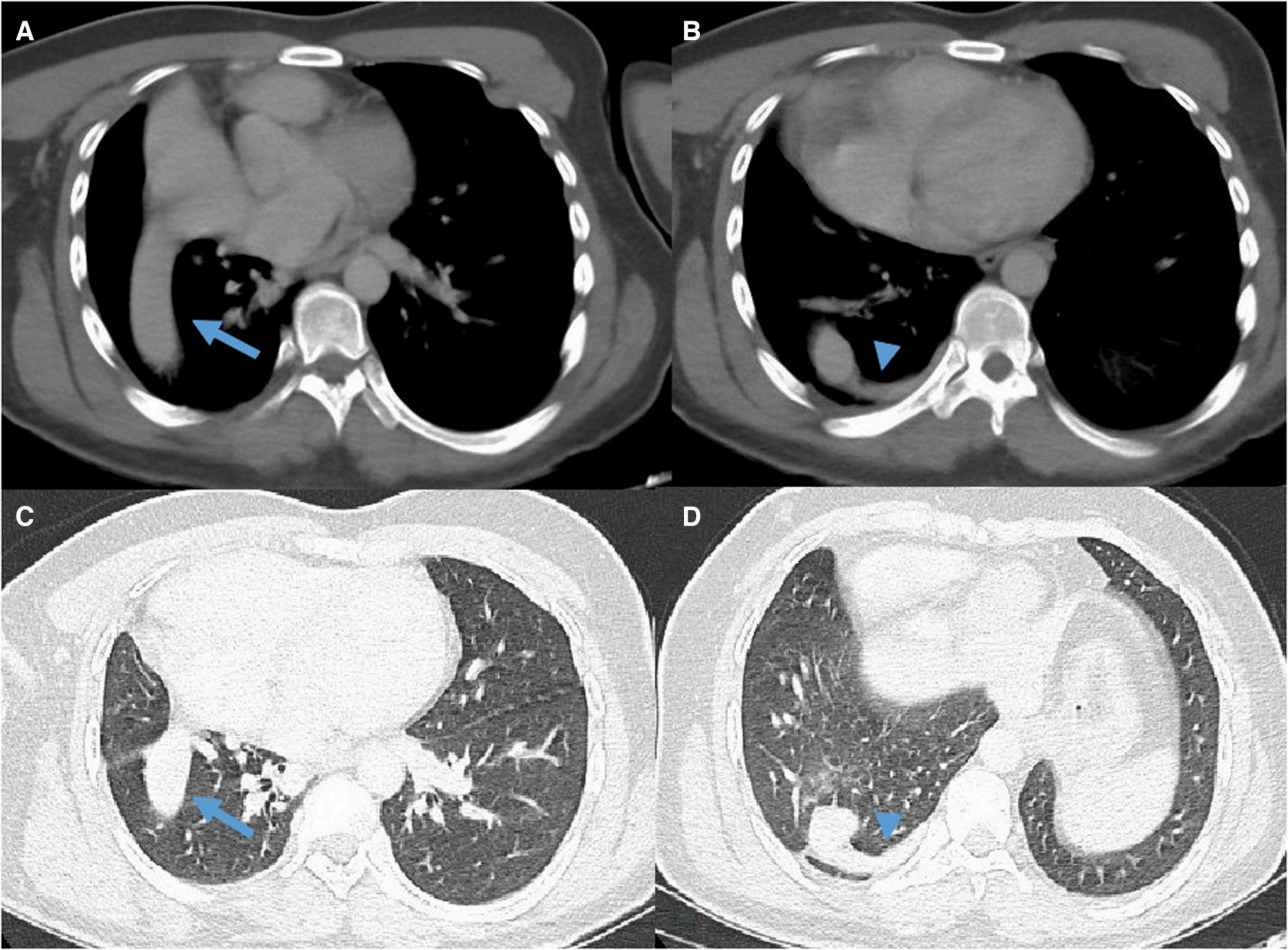
(A, B) mediastinal window; (C, D) lung window. Axial images show the intrapulmonary course of the inferior vena cava (arrow). The azygos vein is connected to the inferior vena cava at the base of the right hemithorax (arrowhead).

The azygos arch was missing, and the azygos vein was linked to the IVC via an arcuate venous duct, located at the base of the right hemithorax (Figure 5A-D).

The patient exhibited situs solitus, and no anomalies consistent with heterotaxy syndrome were evident.

Finally, no further anomalous pulmonary venous returns were detected, and no significant abnormalities were observed in the lung parenchyma.

## Discussion

The inferior vena cava is a venous trunk, formed by the confluence of the right and left common iliac veins, which drains into the right atrium. The tributaries of the IVC include the lumbar veins, the renal veins, the right adrenal vein, the right gonadal vein, and the hepatic veins.

The IVC courses to the right of the aorta within the retroperitoneum and ascends into the thorax through the caval hiatus of the diaphragm, located in the central tendon at approximately the level of the T8 vertebra.^2^^,^^4^^,^^7^

Anatomically, the IVC is commonly divided into suprahepatic, intrahepatic, and infrahepatic segments, the latter further subdivided into suprarenal, renal, and infrarenal portions.^4^^,^^7^

No universally accepted classification of IVC variations exists. Huntington and McClure, in 1920, described up to 14 developmental variants based on studies in domestic cats. The most frequently reported anomalies include retroaortic left renal vein, left-sided IVC, duplicated IVC, circumaortic left renal vein, interrupted IVC with azygos or hemiazygos continuation, absence of the infrarenal IVC, and circumcaval ureter.^1–6^

Embryologically, the IVC is derived from a complex network of embryonic veins, primarily from the right cardinal system, except for its hepatic segment, which originates from the vitelline veins. The renohepatic, renal, and infrarenal segments arise from contributions of the right subcardinal vein, suprasubcardinal anastomoses, right supracardinal vein, and posterior cardinal veins.^8–14^

Developmental anomalies involving the right subcardinal vein, particularly failure of the subcardinal–hepatic anastomosis, lead to interruption of the IVC with azygos or hemiazygos continuation. In these cases, the hepatic segment is not entirely absent and drains directly into the right atrium, while the suprahepatic IVC is absent and the remaining venous flow is redirected through a dilated azygos or hemiazygos vein.^10^^,^^15^^,^^16^

In hemiazygos continuation, the azygos vein is typically hypoplastic, whereas in azygos continuation it is enlarged and arches within the superior mediastinum to drain into the superior vena cava.^4^

The present case demonstrates an unusual course and configuration of the hepatic and suprarenal segments of the IVC. Notably, the vessel does not follow its typical right paraaortic course but traverses the hepatorenal space. It does not pass through the hepatic parenchyma and crosses the diaphragm posteriorly, rather than through the usual caval hiatus. Moreover, the IVC reaches the right atrium through the basal portion of the right lung parenchyma, assuming a configuration reminiscent of the characteristic “scimitar” shape.

Furthermore, the hepatic veins converge into a common venous trunk that drains directly into the right atrium, representing a well-recognized anatomical variant.

An additional notable finding in this case is the absence of the azygos arch, with the presence of an alternative venous structure at the base of the right hemithorax. This feature may have clinical significance, as it implies lack of communication between the superior and inferior vena cava systems.

We conducted an extensive literature review (1990-2024) on IVC anomalies and found no previously reported cases describing an intrapulmonary course of the inferior vena cava. We hypothesize that this unusual anatomical presentation may result from a combination of alterations in the embryological development of the IVC and the azygos system.

Specifically, the failure of the subcardinal–hepatic (vitelline) anastomosis may account for the absence of the hepatic segment, while the persistence of a connection between the right supracardinal vein and the right sacrocardinal segment may explain the anomalous continuation of the infrarenal IVC into the azygos system.^9^^,^^10^^,^^14^^,^^17–19^

Scimitar syndrome is not strictly an IVC anomaly but rather a rare congenital condition characterized by a partial anomalous pulmonary venous return.

Although the morphology observed in this case resembles that seen in scimitar syndrome, the two entities are distinct.

It presents in two main forms: an adult form, typically asymptomatic, and an infantile form, which manifests early with failure to thrive, pulmonary hypertension, heart failure, recurrent pneumonia, and tachypnea. The anomalous vein courses anterior to the right lung hilum, descends inferiorly, and drains into the IVC near the right hepatic vein.^12^

The syndrome is classically characterized by hypoplasia of the right lung, cardiac dextroposition, and pulmonary venous drainage of the right lung into the IVC.

In two-thirds of cases, the scimitar vein is responsible for draining the entire right lung, whereas in the other one-third, it drains only the lower portion of the right lung.^12^

Computed tomography (CT) plays a pivotal role in identifying these anomalies due to its high spatial resolution, multiplanar reconstruction capability, and rapid acquisition time.^5^^,^^20–22^ A delay of 70-90 seconds after contrast administration is recommended to achieve optimal enhancement of the IVC and major venous structures, improving diagnostic accuracy.^5^^,^^23^

In conclusion, although most IVC anatomical variations are asymptomatic,^24^^,^^25^ their recognition through imaging is essential to avoid complications during surgical or interventional procedures and to minimize clinical risks.

## Learning points

- Congenital anomalies of the inferior vena cava (IVC) are often incidentally detected using cross-sectional imaging modalities.
- There is no definite classification of IVC variations. The most common and documented IVC anomalies include retroaortic left renal vein, left IVC, double IVC, circumaortic left renal vein, interrupted IVC with azygos and hemiazygos continuation, absent infrarenal IVC, and circumcaval ureter.
- CT plays a pivotal role in detecting the IVC anomalies, to prevent inadvertent complications during surgical or interventional procedures, and to reduce clinical repercussions.
